# Epstein-Barr virus Peptide Presented by HLA-E is Predominantly Recognized by CD8^bright^ Cells in multiple Sclerosis Patients

**DOI:** 10.1371/journal.pone.0046120

**Published:** 2012-09-25

**Authors:** Pernille B. Jørgensen, Astrid H. Livbjerg, Hans J. Hansen, Thor Petersen, Per Höllsberg

**Affiliations:** 1 Department of Biomedicine, Aarhus University, Aarhus, Denmark; 2 Department of Neurology, MS Clinic, Aarhus University Hospital, Aarhus, Denmark; 3 Danish Neuroscience Center, Aarhus University and Aarhus University Hospital, Aarhus, Denmark; National Institutes of Health, United States of America

## Abstract

Multiple sclerosis (MS) is associated with Epstein-Barr virus (EBV) infection, but impaired immune suppression may be part of the disease pathogenesis. CD8^+^ T cells that are restricted by HLA-E exert an important immunoregulatory mechanism. To explore how EBV might interfere with immune regulation, we examined the expression of HLA-E and the frequency of CD8^+^ cells recognizing HLA-E, presenting either an EBV peptide from the BZLF1 protein or a signal sequence peptide from HLA-A2, in relapsing remitting (MS-RR), primary progressive (MS-PP) MS patients, and healthy controls (HC). Treatment with IFN-α or EBV increased HLA-E expression on CD4^+^ cells. However, only MS-PP had increased expression of HLA-E on resting CD4^+^ cells when compared with HC (p<0.005). CD8^+^ cells were divided into CD8^bright^ and CD8^dim^ cells by flow cytometry analyses. MS-RR had significantly fewer CD8^dim^ cells than HC (p<0.003). Flow cytometry analyses were performed with HLA-E tetramers folded in the presence of the EBV or HLA-A2 peptide to identify HLA-E-interacting cells. MS-RR had increased frequency of CD8^bright^ cells recognizing HLA-E/A2 (p = 0.006) and HLA-E/BZLF1 (p = 0.016). Conversely, MS-RR had fewer CD8^dim^ cells that recognized HLA-E/BZLF1 (p = 0.001), but this could be attributed to the overall lower number of CD8^dim^ cells in MS-RR. Whereas HLA-E/A2 was predominantly recognized by CD8^dim^ cells, HLA-E/BZLF1 was predominantly recognized by CD8^bright^ cells in MS-RR and MS-PP, but not in HC. As expected, HLA-E/A2 was also recognized by CD8-negative cells in a CD94-dependent manner, whereas HLA-E/BZLF1 was poorly recognized in all groups by CD8-negative cells. These data demonstrate that MS-RR patients have expanded their CD8^bright^ cells recognizing HLA-E/BZLF1. Moreover, HLA-E/BZLF1 appears to be recognized by the immune system in a different manner than HLA-E/A2.

## Introduction

Multiple sclerosis (MS) is an organ-specific disease of the central nervous system with an early inflammatory reaction [1]. Environmental, genetic and possibly stochastic events are thought to be important for the development of disease, although the mechanisms are largely unknown. Based on clinical and sero-epidemiological observations, virus infection has been suspected to be involved. Nevertheless, it has been controversial whether or not different viruses could induce a common trigger or whether few or perhaps only one virus would be able to do this.

During the last decade, accumulating data suggest that Epstein-Barr virus (EBV) is strongly and possibly causally linked to MS [2]–[4]. Virtually all MS patients, but not healthy controls, have seroconverted to EBV [5]–[9]. Importantly, 86–99% of children with MS have detectable antibodies to EBV, although only 64–72% of healthy control children have seroconverted at a similar age [10]–[12]. Large prospective database analyses of individuals with infectious mononucleosis have demonstrated a significant increase in later occurrence of MS [13], [14], and prospective sero-epidemiological analyses have shown an increased titer of anti-EBV antibodies in individuals that later went on to develop MS [15]. Moreover, EBV infection is consistent with a number of known epidemiological features of MS [2], [3].

Despite the accumulating evidence of a linkage between EBV infection and MS, the mechanisms of how this infection is linked with the disease remains to be determined. A direct infection of cells within CNS by EBV has been suggested [16] although this remains controversial [17], [18]. In addition, the similarities in pathology between MS and the lesions in animal models of experimental autoimmune encephalomyelitis have suggested an autoimmune component might also be involved in the pathogenesis of MS. Indeed, the importance of the immune system in the progression of the disease is demonstrated by the success of anti-very late antigen (VLA)-4 (CD49d) antibody treatment in MS. Blocking the CD49d integrin prevents recruitment of T cells to the CNS and significantly decreases attack rates in patients [19]. However, treatments that target B cells (anti-CD20) have also been shown to be of benefit in the disease [20], indicating a complex immune mediated activation during the pathogenesis of MS.

A key question is whether MS patients have a defect in their immunosuppressive functions. Immunosuppression is an important mechanism of peripheral tolerance and is mediated by different cell types. One of these cell types is the CD8^+^ regulatory T cell that is restricted by HLA-E. These CD8^+^ cells are important in the development and control of autoimmune disease [21]. In contrast to classical MHC class I molecules, HLA-E is preferentially expressed on activated CD4^+^ T cells [22], although expression can be detected in most tissues. HLA-E is oligomorph with few alleles, and the presentation of peptide on HLA-E may serve different purposes [23]. HLA-E/peptide may engage CD94/NKG2A (inhibitory receptor) or CD94/NKG2C (activating receptor) on NK cells, which typically express no or low levels of CD8 (i.e., CD8 negative or CD8^dim^ cells). Blocking CD94 by a monoclonal antibody allows the identification a small subset of CD8^bright^ T cells that recognizes HLA-E by their T-cell receptor [24]. Presentation of HLA-E/peptide may induce cytotoxicity e.g. by clonal expansion of cytotoxic NK-T cells, which are alloreactive [25], or by eliciting GroEL-specific, cytotoxic CD8^+^ T cells that may cross-react with heat-shock protein (hsp) 60 in stressed, uninfected cells [25]–[27]. HLA-E may also provide a mechanism to suppress the HLA-E-expressing CD4^+^ T cells by HLA-E-restricted CD8^+^ T cells [22], [28]. In this case, the presented peptide may be derived from the β-chain of the T-cell receptor variable region [29]–[32]. Perhaps the most convincing data demonstrating that HLA-E is a restriction element for suppressor CD8^+^ T cells come from studies of Qa-1 in mice, the functional equivalent gene to HLA-E. Qa-1-deficient mice develop exaggerated secondary CD4^+^ T-cell responses after viral infection or immunization with foreign or self peptides [33].

HLA-E-restricted CD8^+^ T cells might be important in MS, since Qa-1-deficient animals have increased susceptibility to recurrence of proteolipid protein (PLP)-induced EAE [33]. Anti-myelin basic protein (MBP)- and anti-myelin oligodendrocyte glycoprotein (MOG)-reactive CD8^+^ T-cell clones restricted by HLA-E have been generated from MS patients, demonstrating that their expression of CD94 and NKG2A was increased during exacerbation, which may limit their capacity to inhibit autoreactive CD4^+^ T cells [34]. It has also been suggested that the treatment of MS patients with glatiramer acetate (Copaxone) in part exerts its effect by inducing cytotoxic CD8^+^ T cells that directly kill CD4^+^ T cells [35].

To explore the mechanisms of how EBV might interfere with immune regulation in relapsing remitting (MS-RR) and primary progressive (MS-PP) MS patients as well as healthy controls (HC), we examined the expression of HLA-E and the frequency of CD8^+^ cells recognizing HLA-E. We made use of HLA-E tetramers presenting either an EBV peptide from the BZLF1 protein or a signal sequence peptide from HLA-A2.

## Materials and Methods

### Culture medium

All cells were cultured with RPMI 1640 (Invitrogen, Taastrup, Denmark) medium supplemented with 10 mM HEPES, streptomycin (0.2 g/l) and glutamine (0.292 g/l), 10% normal human serum (NHS) and 500 IE/ml IL-2 (Proleukin, Nomeco, Copenhagen, Denmark).

### Peripheral blood mononuclear cells

Blood was collected in Venoject vacutainers with citrate (Seelen, Holstebro, Denmark) following informed, written consent from 12 MS-RR and 7 MS-PP and 18 HC, who were all consecutively recruited. The study was approved by the regional ethical committee of Aarhus County (1995/3479). The RMSS group had a mean age of 45 (SD 9; range 27–56) with a female-to-male ratio of 3; the MSPP group had a mean age of 45 (SD 15; range 23–63) with a female-to-male ratio of 0.75; and the HC group had a mean age of 39 (SD 12; range 24–62) with a female-to-male ratio of 0.6. Interface PBMCs were isolated following centrifugation on Ficoll-Paque, washed twice and frozen in 90% FCS+10% dimethyl sulfoxide (DMSO, Sigma-Aldrich, Brøndby, Denmark) for further use. Serum from all individuals was examined for the presence of EBNA1 IgG by ELISA (Biotest, AG, Dreieich, Germany).

Isolation of CD4+ cells from mononuclear cells was performed using magnetic bead separation as described by the manufacturer (Dynal Biotech, Invitrogen, Taastrup, Denmark).

### Cytokine stimulation assays

Cytokine stimulation of HLA-E expression was performed on CD4^+^ T-cell clones that were generated and maintained as previously described [36]. CD4^+^ T-cell clones were incubated in culture medium with 0, 5000 or to completely saturate the receptors 100,000 U/ml IFN-α (PBL Biomedical Laboratories, No. 11100-1) for 0, 4, 7, or 10 hours. After the incubation, cells were fixed with 1% paraformaldehyde for 5 min, centrifuged and resuspended in PBS. Cells were kept in the dark at 4°C until analysis by flow cytometry.

### EBV infection

EBV containing supernatant was harvested from the producer cell line B95-8. PBMC were incubated for 30 hours at 2×10^6^ PBMCs/ml with one half volume of EBV-containing supernatant in the presence or absence of neutralizing anti-human IFN-α antibody (AHC4814, Invitrogen, Taastrup, Denmark) diluted 1∶100. After incubation, the cells were stained with anti-HLA-E and anti-CD4 antibodies for flow cytometry analysis.

### Flow cytometry analysis

Immunofluorescence analyses were performed by incubating the cells in RPMI+10% NHS on ice for 30 min light protected with either antibodies directly conjugated with FITC or PE or by unconjugated primary antibody followed by a secondary conjugated antibody. Antibody concentration was determined by prior titration experiments. Cells were fixed in 1% paraformaldehyde prior to analysis. Expression of HLA-E was determined using anti-HLA-E (MEM-E/07, Abcam, Cambridge, UK) diluted 1∶100 as primary antibody and PE-conjugated rabbit-anti-mouse (R0439, DAKO, Glostrup, Denmark) diluted 1∶10 or FITC-conjugated goat-anti-mouse (F2772, Sigma-Aldrich) diluted 1∶100 as secondary antibody. CD4 expression was determined using FITC-conjugated anti-CD4 (F0766, DAKO) diluted 1∶10. Two homo-tetramers of HLA-E were purchased from Baylor College of Medicine (MHC Tetramer Lab, Houston, TX). The tetramers were created by refolding HLA-E*01033 with the HLA-A2 leader peptide, VMAPRTLVL (referred to as HLA-E/A2), or with the EBV peptide, BZLF-1_39–47_, SQAPLPCVL (referred to as HLA-E/BZLF1). The tetramers were directly labelled with phycoerythrin. Incubations with tetramers diluted 1∶200 (according to the protocol from MHC Tetramer Lab) were done for one hour with anti-CD8 antibody (F0765, DAKO) diluted to final dilution of 1∶10 added 10 min prior to the tetramers, to make sure that the antibody could bind without being sterically hindered by the large tetramers. Likewise, in CD94 blocking experiments, anti-CD94 antibodies (IM1610, Immunotech, Marseilles, France) diluted 1∶10 were added prior to staining with directly conjugated tetramers. Fc receptor binding was reduced by depletion of monocytes by bead extraction according to the manufacturer's protocol (Dynabeads CD14, Invitrogen). Samples were analyzed on a FC500 flow cytometer (Beckman Coulter, Miami, FL) by collecting 10,000 events when staining with antibodies alone or 100,000 events when staining was performed with tetramers. All samples were gated on lymphocytes using forward and side light-scatter parameters, and data were analyzed using FlowJo software (Tree Star inc., San Carlos, CA).

### Western blotting

For western blotting analysis, cells were lysed for 25 min in ice-cold lysis buffer (Cell Signaling, #9803) supplemented with 1.0 mM PMSF (Sigma), 5.0 mM NaF and 150 µl Complete Mini protease inhibitor cocktail (Roche, Hvidovre, Denmark). The lysates were then centrifuged at 2000×*g* for 5 min at 4°C to remove large cell debris and again at 16000×*g* for 10 minutes at 4°C. Lysates were frozen at −70°C until they were analyzed by Western blotting. Protein concentration was determined by Bio-Rad Protein Assay (Bio-Rad Laboratories, Inc., Hercules, CA) and 30 µg protein was added per lane on a Criterion XT Bis-Tris 10% gel (Bio-Rad). Proteins were separated under reducing conditions in MOPS buffer (Bio-Rad) and transferred to a nitrocellulose membrane (Amersham, Hillerød, Denmark) in 0.025 M Tris-HCl, 0.192 M Glycin, 20% v/v ethanol (pH 8.3) at 300 mA for 1.5 hours. Equal loading was assured by examining transferred protein in Ponceau-S (Sigma-Aldrich). To reduce non-specific binding, the nitrocellulose was incubated for 1 hour in 5% non-fat dry milk in TBS (0.01 M Tris-HCl; 0.14 NaCl; pH 7.4)+0.1% Tween 20 (Sigma) (TBST). Primary anti-HLA-E antibody (Abcam, MEM-E/02) was diluted 1∶750, according to the manufacturer's protocol. After 1 hour incubation at room temperature, the blot was washed 3 times 5 min in TBST and probed 1 hour at room temperature with the secondary horse radish peroxidase (HRP)-conjugated antibody diluted 1∶2000 in 5% non-fatty milk in TBST. In addition to Ponceau-S staining, another loading control was made by probing with anti-GAPDH antibody (sc-25778, Santa Cruz Biotechnology, Santa Cruz, CA) diluted 1∶200 according to the manufacturer's protocol. Blots were developed using enhanced chemiluminescence SuperSignal West Pico Chemilumiscent Substrate (Pierce, Rockford, IL). Negative control lysates were derived from the mouse RMA-S cells, which lack HLA-E expression, and positive control lysates were derived from RMA-S cells transfected with human beta-2 microglobulin and HLA-E (RMA-S-EM7). These cell lines were a kind gift from Dr. Elisabeth Weiss, Germany [37].

### Statistical analysis

Statistical analysis was performed using two-tailed Mann-Whitney U-test. Paired data with anti-CD94 blocking was analysed by two-tailed, paired t-test. Significant difference was defined as a p-value<0.05.

## Results

### Expression of HLA-E on resting CD4^+^ cells from MS patients

**Figure 1:**
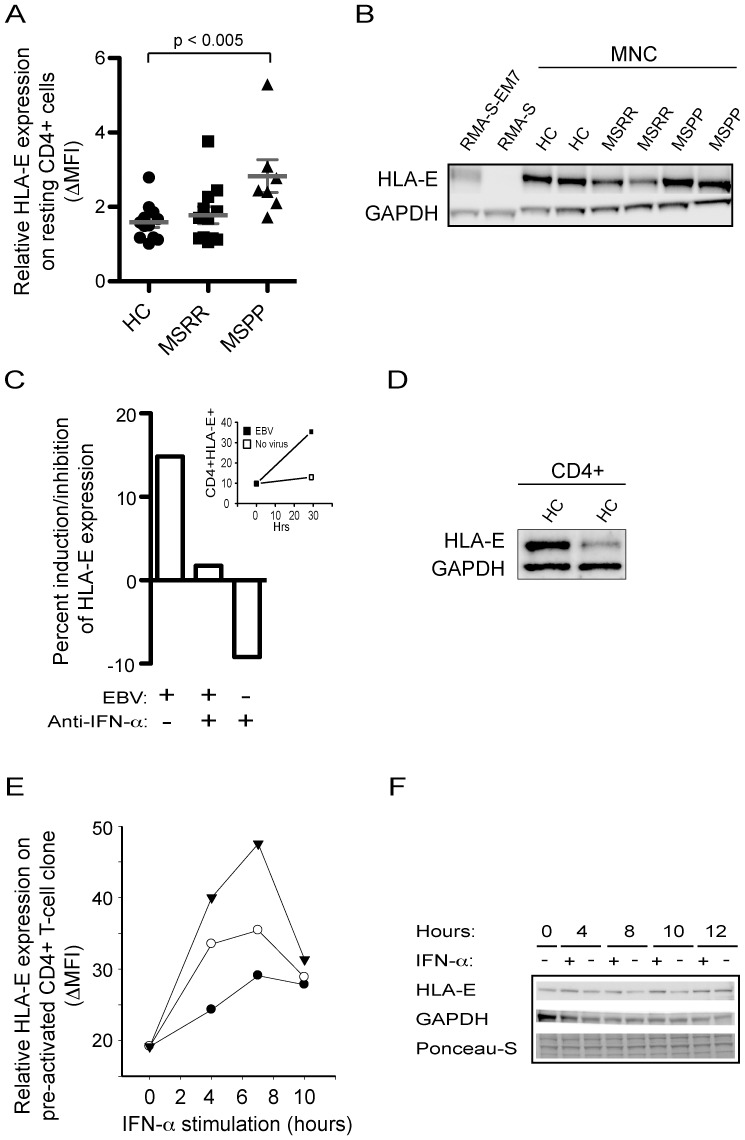
HLA-E expression. (A) Primary progressive MS patients have enhanced expression of HLA-E on CD4^+^ cells. Mononuclear cells from healthy controls (HC), relapsing-remitting MS patients (MS-RR) and primary progressive MS patients (MS-PP) were incubated with fluorophore-conjugated antibodies against HLA-E and CD4 and analysed by flow cytometry. Results are expressed as median of fluorescence intensity. (B) Expression of HLA-E on mononuclear cells from patients and controls. Western blot analysis of HLA-E expression on lysates from mononuclear cells of healthy controls and MS patients. Positive and negative control lysates are from RMA-S-EM7 and RMA-S cells. GAPDH is used as a loading control. Results are representative of two experiments. (C) EBV induces expression of HLA-E. Mononuclear cells were treated with EBV containing supernatant from B95-8 or medium in the presence or absence of neutralizing anti-IFN-α antibody. Results are expressed as induction/inhibition in percentage of uninduced cells by calculating [(MFI_treated_−MFI_untreated_)/MFI_untreated_]×100%. Insert shows flow cytometric analyses of CD4^+^HLA-E^+^ on the mononuclear cells at 0 hr and 29 hrs incubation with EBV. Results are representative of 2 independent experiments. (D) Expression of HLA-E on CD4^+^ cells. Western blot analysis of HLA-E expression on lysates from CD4^+^ cells isolated from mononuclear cells by magnetic bead separation. GAPDH is shown as a loading control. (E) IFN-α induces surface-expression of HLA-E. A CD4^+^ T-cell clone was stimulated between 0 and 10 hours in the presence or absence of IFN-α. Cells were incubated in the absence (closed circles) or presence of 500 U/ml (open circles) or 100,000 U/ml of IFN-α (triangles). Cells were examined by flow cytometry and results expressed as Δ median fluorescence intensity (ΔMFI) of HLA-E expression when incubated with anti-HLA-E antibody minus the MFI of an isotype control antibody. Results are representative of 5 independent experiments. (F) IFN-αinduces expression of HLA-E. Cells were lysed at the indicated time and analyzed by Western blotting. Nitrocellulose was probed with anti-HLA-E and as a loading control an anti-GAPDH antibody. Ponceau-S staining of the nitrocellulose indicates even transfer of proteins between the lanes.

To address a possible role of HLA-E in MS, we initially examined its surface expression on resting CD4^+^ cells from healthy controls (HC), relapsing-remitting (MS-RR) and primary progressive (MS-PP) MS patients (Fig. 1A). Expression of HLA-E was generally low, but significantly increased on CD4^+^ cells from primary progressive MS patients when compared with healthy controls (p<0.005) and relapsing remitting MS patients (p<0.031). Both patients and controls expressed HLA-E as determined by Western blotting (Fig. 1B)

### EBV-induced HLA-E expression by an IFN-α-dependent pathway

EBV exposure or infection may induce interferons [33]; [34]. To examine whether EBV was able to induce HLA-E expression, mononuclear cells were incubated with EBV supernatant for 30 hours and the percentage of cells expressing HLA-E was measured at an intensity above a cut-off level where only 1% of cells stain with isotype control. Addition of EBV enhanced the number of HLA-E positive mononuclear cells by approximately 15% (Fig. 1C) and among these, CD4^+^ cells expressed HLA-E (Fig. 1D) and demonstrated upregulated HLA-E expression upon EBV treatment (Fig. 1C, insert). Some of this induction may be attributed to the secretion of IFN-α, since the number of HLA-E positive mononuclear cells reverted to the level of uninduced cells in the presence of EBV and anti-IFN-α antibody. However, anti-IFN-α inhibited the expression of HLA-E in the absence of EBV indicating, that EBV may also induce HLA-E by a mechanism separate from IFN-α. Nevertheless, IFN-α may be a relevant inducer of HLA-E expression during virus infection.

### Induction of HLA-E by IFN-α

HLA-E molecules are expressed at high levels in pre-activated CD4^+^ T cells, such as CD4^+^ T-cell clones. To further study the induction of HLA-E, we measured by flow cytometry the ability of type I and II interferons to induce surface expression of HLA-E. Since IFN-γ induces HLA-E gene transcription, we first confirmed that IFN-γ induced expression of HLA-E in our system. As expected, the presence of 40, 80, or 160 ng/ml of IFN-γ induced a dose-dependent increase in HLA-E expression on isolated CD4^+^ T-cell clones 4 hours after addition of the cytokine (data not shown). To examine whether IFN-α was capable of inducing HLA-E expression, isolated CD4^+^ T-cell clones were incubated in the absence or presence of recombinant IFN-α for 4, 7, and 10 hours and the expression of HLA-E was measured by the median fluorescence intensity on flow cytometry. Recombinant IFN-α induced expression of HLA-E by addition of 500 U/ml, an induction that could be further enhanced by the presence of very high concentration of IFN-α (Fig. 1E). The presence of IFN-α also increased the total level of HLA-E molecules as measured by Western blotting analysis (Fig. 1F).

### Recognition of HLA-E/A2 and HLA-E/BZLF1 by different CD8 cell subsets

**Figure 2:**
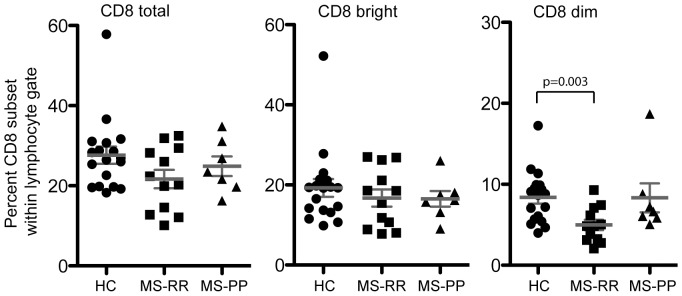
MS-RR patients have reduced percentage of CD8^dim^ cells. Mononuclear cells from HC, MS-RR, and MS-PP was incubated with fluorophor-conjugated anti-CD8 antibody and examined by flow cytometry for the presence of CD8 total, CD8^bright^, and CD8^dim^ cells. Comparisons are done using Mann-Whitney U-test.

HLA-E/peptide complexes may be recognized by either CD94/NKG2A/C (predominantly CD8 negative or CD8^dim^ cells) or by the T-cell receptor (predominantly CD8^bright^ cells). Therefore, the distribution of these CD8 cells was first characterized (Fig. 2). Although the frequency of CD8^+^ (total) cells and CD8^bright^ cells were similar in HC, MS-RR and MS-PP, the presence of CD8^dim^ cells was significantly reduced among the MS-RR patients (p = 0.003).

We then examined the binding to CD8^+^ cells of two HLA-E tetramers that presented either an endogenous peptide from the HLA-A2 leader sequence (HLA-E/A2) or an exogenous peptide derived from EBV (HLA-E/BZLF1). These tetramers represented the most frequently found type of peptide (i.e. from the signal sequences of HLA class I) and a peptide only expressed during cellular stress (infection or reactivation of EBV). All patients and all HC but one were positive for EBNA1 IgG by ELISA.

**Figure 3:**
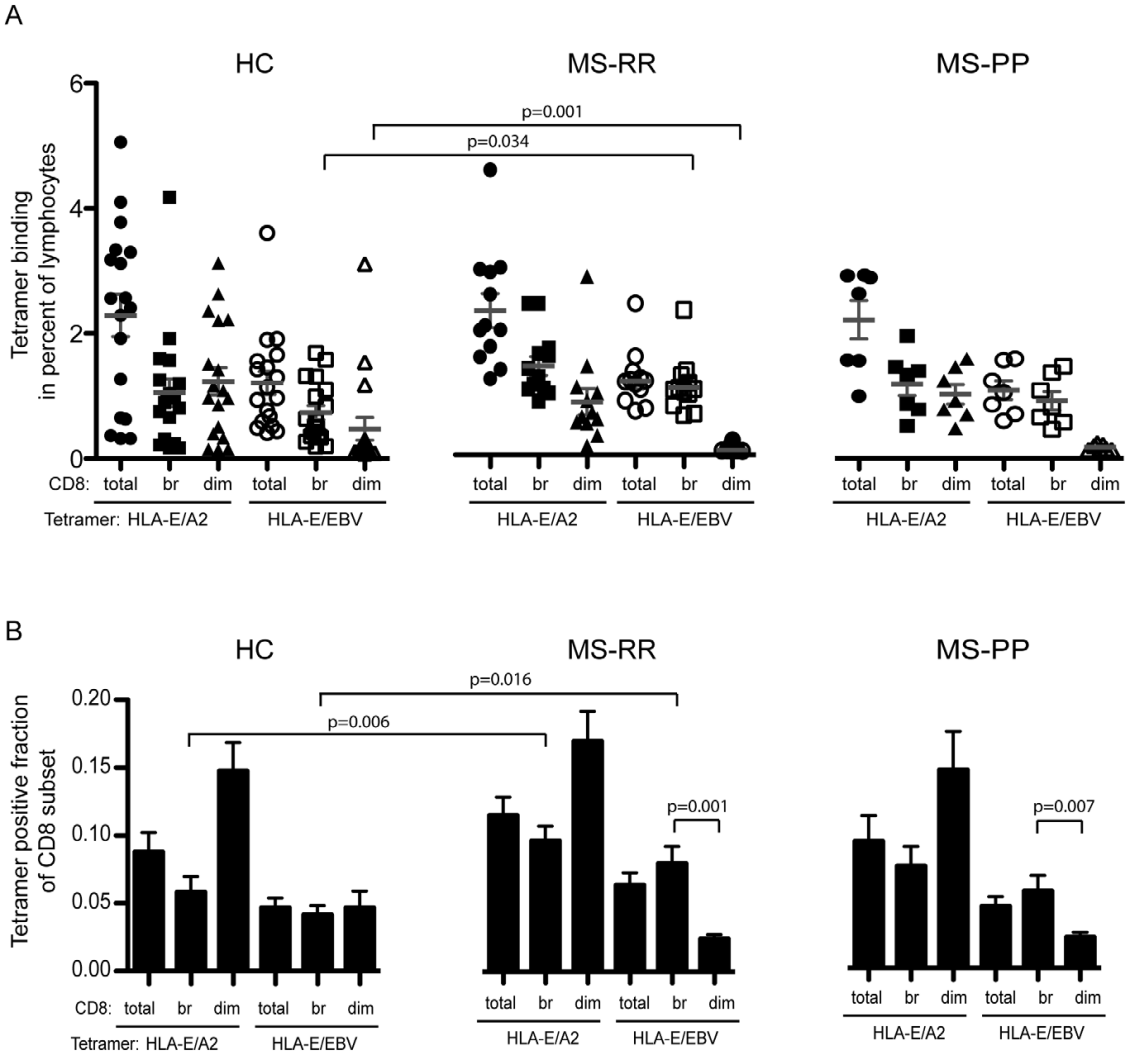
Increased frequency of cells recognizing HLA-E/BZLF1 by CD8^bright^ cells in MS-RR patients. (A) Mononuclear cells from HC, MS-RR, and MS-PP were incubated with tetramers of HLA-E/A2 and HLA-E/BZLF1 and anti-CD8 antibody. Tetramer positive cells are expressed as percentage of cells binding the tetramer within the lymphogate for CD8 total, CD8^bright^, and CD8^dim^ cells. Mean ± SEM is indicated (B) The frequency of tetramer positive cells are expressed as a fraction of the indicated CD8 subset to correct for altered number of CD8^+^ cells between the groups. SEM is indicated on the top of the bars. Comparisons are done using Mann-Whitney U-test.

Overall, the frequency of cells recognizing HLA-E/A2 was higher than the frequency of cells recognizing HLA-E/BZLF1. However, when divided into the CD8 total, CD8^bright^ and CD8^dim^ subset, MS-RR had a significantly reduced binding of HLA-E/BZLF1 to the CD8^dim^ subset (p = 0.001) when compared with HC, in contrast to the other cell subsets examined. Notably, the MS-RR patients had an increased recognition of HLA-E/BZLF1 by their CD8^bright^ cells (p = 0.034) when compared with HC (Fig. 3A). Since the frequencies of the subsets were different between HC and MS-RR, the data were corrected for this by expressing the results as fraction of tetramer positive cells within the CD8^+^ cells (Fig. 3B). This indicated that the reduced recognition of HLA-E/BZLF1 by CD8^dim^ cells could in part be attributed to fewer CD8^dim^ cells (the difference was no longer statistical significant), whereas the increased occurrence of CD8^bright^ cells recognizing HLA-E/BZLF1 was even more pronounced after this correction (p = 0.016). The correction also indicated an increased fraction of CD8^bright^ cells recognizing the HLA-E/A2 tetramer (p = 0.006). For both the MS-RR and MS-PP, the fraction of CD8^dim^ cells was significantly lower than the CD8^bright^ cells. This was not the case for HC (Fig. 3B). Together this indicated an altered recognition of HLA-E/peptide by MS-RR and MS-PP patients.

**Figure 4:**
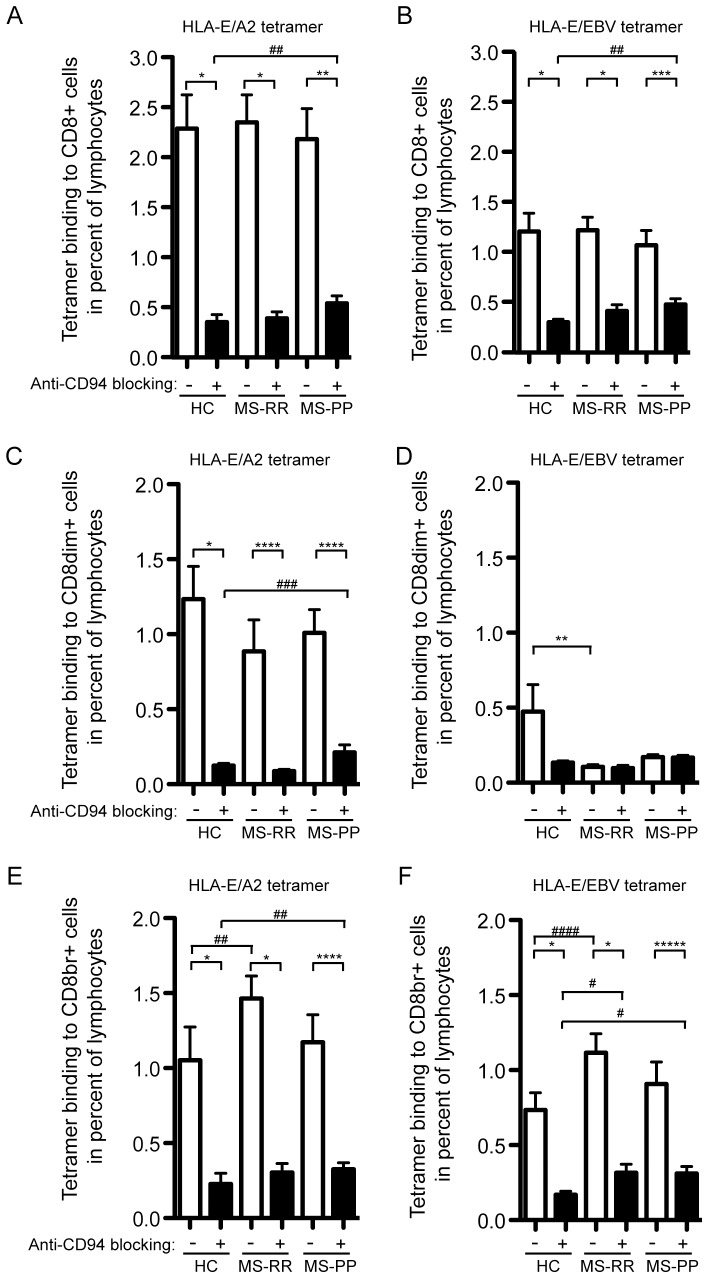
Increased frequency of cells recognizing HLA-E/BZLF1 by CD8^bright^ cells in a CD94-independent manner in MS-RR and MS-PP patients. Tetramer HLA-E/A2 binding to CD8 total (A), CD8^bright^ (C), and CD8^dim^ (E) and tetramer HLA-E/BZLF1 binding to CD8 total (B), CD8^bright^ (D), and CD8^dim^ (F) are all expressed as percentage binding within lymphogate. Binding is analysed in the presence or absence of anti-CD94 blocking antibody. Comparisons between presence and absence of anti-CD94 are done using paired T-test. Other comparisons are done using Mann-Whitney U-test. The symbols indicating different p values are: * p≤0.0001, ** p≤0.001, *** p≤0.005, **** p≤0.003, ***** p≤0.007, # p≤0.01, ## p≤0.02, ### p≤0.03, #### p≤0.04.

The CD8^bright^ cells are thought to predominantly represent CD8^+^ T cells, whereas CD8^dim^ cells are predominantly NK cells, or CD8^+^ T cells expressing alpha-alpha CD8 homodimers [38]. Although both T cells and NK cells may express the primary recognition molecule for HLA-E, CD94, blocking of CD94 may help elucidating the recognition of HLA-E by the T-cell receptor. This may be important, since cells recognizing HLA-E by the T-cell receptor complex may have different functions than cells recognizing HLA-E by the CD94 complex. When anti-CD94 was added to the cells, the recognition of the tetramer was uniformly reduced (Fig. 4A, B, C, D, F). The exception was CD8^dim^-cell recognition of HLA-E/BZLF1 in MS-RR and MS-PP, which was very low even without anti-CD94 (Fig. 4D). This may indicate that HLA-E/BZLF1 is poorly recognized by NK cells. In agreement with this notion, the recognition of HLA-E/BZLF1 was clearly found in the CD8bright population, where the frequency of HLA-E/BZLF1 tetramer positive cells was comparable to HLA-E/A2 positive cells. Importantly, after blocking CD94, the remaining population of CD8^bright^ cells recognizing HLA-E/BZLF1 was significantly higher in MS-RR patients when compared with HC (p≤0.01) (Fig. 4F). The similar comparison for recognition of the HLA-E/A2 complex was insignificant between these two groups (Fig. 4E), whereas MS-PP has significantly increased frequency of CD8^bright^ cells recognizing either tetramer when compared with HC.

**Figure 5:**
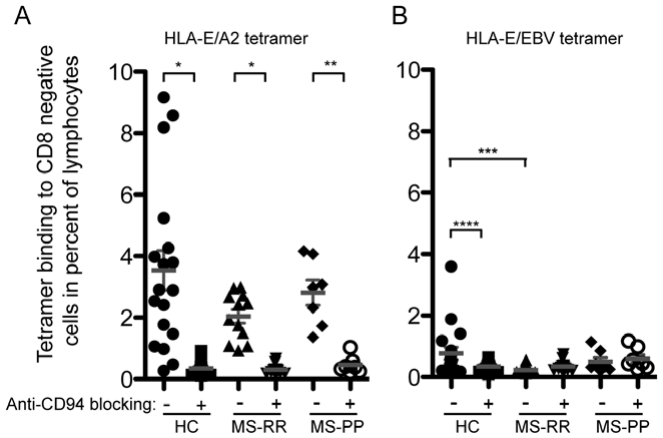
Decreased binding of HLA-E/BZLF1 to CD8-negative cells in MS-RR patients. Tetramer binding of HLA-E/A2 (A) and HLA-E/BZLF1 (B) to CD8-negative cells is shown in percentage of lymphocytes (lymphogate). Binding is analysed in the presence or absence of anti-CD94 blocking antibody. Comparisons between presence and absence of anti-CD94 are done using paired T-test. Other comparisons are done using Mann-Whitney U-test. The symbols indicating different p values are: * p≤0.0001, ** p≤0.0008, *** p≤0.0017, **** p≤0.048.

Since the data suggested that HLA-E/BZLF1 was poorly recognized by NK cells, we examined the direct frequency of tetramer positive CD8-negative cells. Recognition of HLA-E by CD8-negative cells is thought to represent interaction with CD94/NKG2A/C on NK cells. In agreement with the above observation, CD8-negative cells predominantly bound HLA-E/A2 and this was completely inhibited by anti-CD94 antibodies (Fig. 5). In contrast, HLA-E/BZLF1 binding was much lower, and almost absent in the MS-RR and MS-PP groups. This further indicated that CD8^+^ T cells and not NK cells recognized HLA-E/BZLF1.

## Discussion

Accumulating evidence suggest that EBV is associated with development of MS [2], [3], [39]. One, yet controversial, possibility is that EBV directly infects cells of the CNS, thereby provoking an immune reaction to virally presented peptides [16]–[18], [40]. Another, and not necessarily mutually exclusive, possibility is that EBV infection participates in perturbation of the immunoregulatory network that is responsible for controlling autoreactive immune responses. HLA-E-restricted CD8^+^ T cells are one of the suppressive cell types responsible for controlling autoreactive CD4^+^ T cells, but whether CD8^+^ T cells from MS patients recognize EBV presented by HLA-E has not previously been addressed.

HLA-E is the least polymorphic of all MHC class I proteins and has two predominant alleles in the Caucasian population. These alleles differ by only one amino acid at position 107 (arginine or glycine) giving rise to HLA-E*0101 (HLA-E^107R^) and HLA-E*0103 (HLA-E^107G^). Of these HLA-E^107G^ is expressed at significantly higher levels than HLA-E^107R^ on normal cells due to higher peptide binding abilities and stability on the cell surface. Thus, our tetramers used the HLA-E*0103/peptide complex for optimal recognition of the cells. We used a protocol from the MHC Tetramer Lab suggesting 1∶200 dilution of the tetramer for staining. However, we cannot exclude the possibility that our staining was below saturation, since higher levels of binding was achieved at higher tetramer concentration. However, higher concentrations of tetramer might also cause higher levels of non-specific tetramer binding. Attempts to compete with HLA-E/BZLF1 binding indicated that 28-fold excess of unlabelled tetramer was needed to obtain more than 50% inhibition. This may indicate that the frequencies of tetramer binding cells are underestimated in our analyses.

The HLA-E/peptide complex may serve as a ligand for CD94/NKG2A or C, which are NK cell inhibitory and activating receptors. CD94/NKG2A appears to bind ligand with higher affinity, which has led to the notion that HLA-E/peptide may protect the cell from NK-cell mediated killing [41]. However, association of other proteins with HLA-E may destroy the interaction with CD94/NKG2A. Stress-induced expression of uncommon peptides on HLA-E may therefore prevent the inhibitory signal and target the cell for NK-cell mediated lysis. An example of this is the HLA-E presentation of a peptide from leader sequence of hsp60, which makes the HLA-E complex unrecognizable for the CD94/NKG2A inhibitory heterodimer [42]. Presentation of the EBV BZLF1_39–47_ peptide by HLA-E may have similar consequences. In contrast to the HLA-E/A2 complex, we found a poor recognition of HLA-E/BZLF1 by CD8-negative cells, which include a large fraction of NK cells. Thus, we propose that HLA-E/BZLF1 may also have impaired affinity for CD94/NKG2A. Interestingly, the recognition of HLA-E/BZLF1 by CD8-negative cells was significantly reduced in the MS-RR group. We did not examine the proportion of NK cells within the lymphocytes in this study, so an explanation for the reduced recognition could be a reduced number of NK cells, which of course in itself might be interesting.

Within recent years it has become clear that HLA-E/peptide is also recognized by CD8^+^ T-cells receptors in a manner comparable to peptides presented by classical MHC class I molecules [32], [43]. In particular, HLA-E-restricted CD8^+^ T-cell recognition of peptides from pathogens may be an important mechanism of immune regulation during infections, as was first demonstrated for *Mycobacterium tuberculosis* [44], [45]. Indeed, peptides from several bacteria and viruses have been shown to bind to HLA-E [23], [46]. Given the association between EBV and MS, we were interested in examining whether MS patients had a detectable CD8^+^ cell-response to the HLA-E/BZLF1 peptide derived from the BZLF-1_39–47_ [47], which has been shown to be recognized by a CD8^+^ T-cell clone [48].

We demonstrate that CD8^+^ cells recognize HLA-E/BZLF1 in all the tested groups. Importantly, HLA-E/BZLF1 is recognized differently from HLA-E/A2. Whereas HLA-E/A2 is predominantly recognized by CD8-negative cells (subset of NK cells) in a CD94-dependent manner, HLA-E/BZLF1 is predominantly recognized by CD8^bright^ cells. These are expected to be T cells. The CD94 blocking precludes binding of the vast majority of HLA-E/A2 to both CD8^+^ and CD8-negative cells. Although HLA-E/BZLF1 did bind CD8^+^ cells in a CD94-independent manner, some of the binding is clearly CD94-dependent. CD94/NKG2 is the most prevalent NK-inhibitory receptor present on activated CD8^+^ T cells and it is possible that some of the CD8^+^ cell binding is through T-cell expressed CD94/NKG2 heterodimers. The HLA-E/BZLF1 tetramer binds poorly to the CD8-negative cells, although this subset is expected to contain an NK cell subset expressing CD94/NKG2. Nevertheless, some HC individuals apparently do have CD94-dependent binding to HLA-E/BZLF1 in the CD8-negative population.

MS-RR patients had normal levels of HLA-E on their CD4^+^ cells, whereas MS-PP had a slight increase in HLA-E expression when compared with HC. A number of factors may impact on HLA-E expression, most notably IFN-γ [49]. We demonstrate that EBV and IFN-α, a cytokine that is induced by EBV and other viral infections [50], [51], are also able to upregulate HLA-E. However, we do not know the mechanism for the altered expression of HLA-E on CD4^+^ cells from MS-PP patients. Our number of MS-PP patients is small and this finding should be reproduced on a larger cohort of MS-PP patients, although we do recognize that these patient samples are difficult to collect, since the MS-PP form of the disease only constitutes 10–15% of all MS patients. In addition, the antibody for HLA-E (MEM-E/07) is known to cross-react with HLA-B7 and to a lesser extent with other classical HLA class I molecules. We did not have information of the haplotype of our individuals, and therefore cannot assess the potential significance of this.

HLA-E/A2 and HLA-E/BZLF1 appeared to be recognized differently. The slight increase in the frequency of CD8^+^ cells that recognize HLA-E/BZLF1 in MS patients may reflect an increased expression of EBV in these patients. Patients latently infected with *Mycobacterium tuberculosis* have expanded their HLA-E-restricted CD8^+^ T cells, which in some patients constitute the dominant CD8^+^ T cell response against the pathogen [44]. CD8^+^ T cells interacting with EBV-infected cells have been demonstrated in the CNS [16], [40]. It may be interesting to examine whether these cells are restricted by HLA-E, that is, whether they will bind the HLA-E/BZLF1 tetramer in a CD94-independent manner. Nevertheless, it is possible that more EBV peptides may bind to HLA-E during infection. A careful examination of HLA-E-restricted responses to peptides from *Mycobacterium tuberculosis* found CD8^+^ T-cell responses to a number of these peptides [45].

Although we have characterized differences in the frequency of HLA-E/peptide reacting cells, we do not know whether they do suppress CD4^+^ T cells. Moreover, we speculate that regulatory CD8^bright^ cells are expanded by an increased expression of EBV in MS patients, but we also suggest that this could in part be due to insufficient elimination of these cells by e.g. NK cells (CD8-negative and CD8^dim^ cells). If B cells were the major presenting cell of HLA-E/BZLF1, this would argue that MS patients might be deficient in controlling these cells. Indeed, elimination of B cells is known to be an effective therapy in MS-RR. Thus, future experiments may address more directly the role of NK cells in controlling the B cell function in MS.
